# Methicillin-resistant S*taphylococcus aureus *prevalence: Current susceptibility patterns in Trinidad

**DOI:** 10.1186/1471-2334-6-83

**Published:** 2006-05-05

**Authors:** Fitzroy A Orrett, Michael Land

**Affiliations:** 1 The Department of Paraclinical Sciences, Faculty of Medical Sciences, University of the West Indies, Eric Williams Medical Sciences, Complex Champs Fleurs, Trinidad and Tobago, West Indies; 2 Department of Biological, Sciences, Northwestern State University, Natchitoches, LA 71497, USA

## Abstract

**Background:**

Methicillin-resistant *Staphylococcus aureus *(MRSA) has become one of the most widespread causes of nosocomial infections worldwide. Recently, reports have emerged that *S. aureus *strains recovered from community-acquired infections are also methicillin-resistant. This study was undertaken to analyze the prevalence of methicillin resistance among isolates at a regional hospital in Trinidad, and document the current resistance profile of MRSA and methicillin-sensitive *Staphylococcus aureus *(MSSA) to the commonly used anti-staphylococcal agents.

**Methods:**

Over a 6-year period we analyzed 2430 isolates of *S. aureus *strains recovered from various clinical sources, from hospital and community practices. Antimicrobial susceptibility testing was done according to guideline recommendations of the National Committee for Clinical Laboratory Standards.

**Results:**

The prevalence of MRSA from surgical/burn wounds, urine and pus/abscess were 60.1%, 15.5% and 6.6%, respectively. The major sources of MSSA were surgical/burn wounds, pus/abscess and upper respiratory tract specimens with rates of 32.9%, 17.1% and 14.3%, respectively. The greatest prevalence of resistance of MRSA was seen for erythromycin (86.7%), and clindamycin (75.3%). Resistance rates among MSSA were highest for ampicillin (70%). Resistance rates for tetracycline were similar among both MRSA (78.7%) and MSSA (73.5%). The MRSA recovery rates from nosocomial sources (20.8%) was significantly higher than that of previous years (12.5%) (p < 0.001), whereas rates among community isolates were relatively similar for the same period (4.1% versus 8.1%).

**Conclusion:**

The prevalence of MRSA in the hospital increased from 12.5% in 1999 to 20.8% in 2004. Most isolates were associated with infected surgical/burn wounds which may have become infected via the hands of HCPs during dressing exercises. Infection control measures aimed at the proper hand hygiene procedures may interrupt the spread of MRSA. HCPs may also be carriers of MRSA in their anterior nares. Surveillance cultures of both patients and HCPs may help to identify carriers who would be offered antibiotics to eradicate the organisms. Most MRSA are resistant to several non-β-lactam antibiotics. Frequent monitoring of susceptibility patterns of MRSA and the formulation of a definite antibiotic policy maybe helpful in decreasing the incidence of MRSA infection.

## Background

*Staphylococcus aureus *remains one of the most frequently isolated pathogens in both community and hospital practices. The organism has been found to be the most common bacterial agent recovered from blood stream infections, skin and soft tissue infections, pneumonia and hospital-acquired post-operative wound infections [1-4]. If untreated, staphylococcal infections may lead to bacteremic seedings of several organs, causing endocarditis, osteomyelitis and septic arthritis [5,6]. Changes in the pattern of antimicrobial susceptibility of *S. aureus *and other organisms have been reported world wide, especially in developing countries [7-9], making antimicrobial agents increasingly less effective in treating bacterial infections.

Over the past twelve years there have been dramatic changes in the susceptibility of *S. aureus *in both hospitals and community settings in Trinidad [10-12]. The older β-lactams, penicillin and ampicillin are ineffective against more than 80 % of isolated strains, and resistance to many of the non-β-lactam agents such as the tetracyclines, gentamicin, chloramphenicol, erythromycin and clindamycin has gradually increased and reached alarming levels by the 1990s in many parts of the world [13-15].

Several mechanisms for the methicillin resistance seen in *S. aureus *have been elucidated. The most important is the production of a unique penicillin-binding protein (PBP) that has a low affinity for β-lactam antibiotics and whose effects are determined by several structural genes (*mec, mec RI, mec I*) [16,17]. Other known mechanisms of methicillin resistance are the production of the usual PBPs, but with modified affinities for the β-lactam drugs, and the hyper production of penicillinase enzyme [17,18].

Methicillin-resistant *Staphylococcus aureus *(MRSA) and methicillin-sensitive *Staphylococcus aureus (MSSA) *strains can easily spread from infected patients to medical staffs, who often become transient carriers [19]. Because MRSA, are usually also resistant to other non-β-lactam antibiotics, infections with them are life-threatening in immunocompromised patients, often difficult to manage, and problematic to eradicate. The primary importance is to decrease the prevalence of MRSA by measures such as rapid and reliable identification of the organisms along with their susceptibility patterns to other antibiotics, isolation and treatment of patients and carriers, and strict adherence to proper hand washing practices by health care providers.

The purpose of this study was to review and document: (1) the current prevalence of methicillin resistance among isolates of *S. aureus *recovered from hospital and community sources and (2), the pattern of antimicrobial susceptibilities of MSSA and MRSA isolates to the commonly prescribed antibiotics in Trinidad.

## Methods

Between January 1, 1999 and December 31, 2004, we examined strains of *S. aureus *recovered from various clinical sources from both hospital and community practices in Trinidad, which had complete susceptibility data. Specimens were derived from patients on the wards and from those attending outpatients' clinics at the Eric Williams Medical Sciences Complex (EWMSC). The EWMSC is a 560-bed semi-private medical facility located in the northwest part of the country. Trinidad is the larger of the twin-island Republic, Trinidad and Tobago, located about 11 km off the northern coast of Venezuela in South America. The population of the Republic is about 1.3 million. For this investigation, no differentiation was between isolates from inpatients and outpatients, all being classified as "hospital practice" isolates. *S. aureus *recovered from patients attending health centers in the community and those seen by general practitioners were classified as "community practice" isolates. For purposes of gathering infection control surveillance data, other organisms and duplicates of clinically significant isolates were excluded from the study.

*S. aureus *isolates were identified via colonial morphology on blood agar plates supplemented with 5% sheep blood, gram stain characteristics, catalase test, tube coagulase test, mannitol fermentation and DNAse test. Antimicrobial susceptibility testing was done on Mueller-Hinton agar (BBL Microbiology Systems, Cockeysville, Maryland, USA) using the disc diffusion technique as outlined by the National Committee for Clinical Laboratory Standards [20]. The following drugs and concentrations (in brackets) were used to determine the antibiogram of the strains: ampicillin (10 μg), gentamicin (10 μg), erythromycin (15 μg), tetracycline (30 μg), co-trimoxazole (trimethoprim-sulfamethoxazole) (1.25/23.75 μg), ciprofloxacin 5 μg), clindamycin (2 μg) and vancomycin (30 μg). Methicillin-resistance was tested using a 1 μg oxacillin disc. Zone diameters were read after incubation at 35°C for a full 24 hr. Strains with zone sizes of < 10 mm for oxacillin was regarded as methicillin resistant. ATCC *S. aureus *strains 25923 and 29213 were used as quality control. Statistical analysis of data was done using X^2 ^test with the Statistical Package for the Social Sciences, version 9.0.

## Results

During the six-year study period, a total of 2430 *S. aureus *isolates were recorded. The major sources of *S. aureus *were from surgical and burn wounds, pus/abscess, upper respiratory tract and urine which together, accounted for 1805 (74.3%) of all isolates (Table 1). One hundred and nine *S. aureus strains *(4.5%) were from patients with pneumonia, 201 (8.3%) from the blood of patients with sepsis and 95 (4.0%) from patients with eye infections. The rest of the isolates were from vaginal, ear, CNS and miscellaneous infections, each accounting for < 3.0% of the total. The major source of MRSA was surgical/burn wounds (60.1%) followed by urine (15.5%), pus/abscess (6.6%) and blood (6.2%). MSSA was recovered more frequently from surgical/burn wounds (32.9%), pus/abscess (17.1%) and the upper respiratory tract (14.3%).

**Table 1:** Clinical sources 2430 strains of *Staphylococcus aureus *isolated at the Eric Williams Medical Sciences Complex, 1999 – 2004

			^a^MRSA		^b^MSSA	
			
Source/Specimen	N	%	N	%	N	%
Wounds (surgical/burns	922	37.9	271	60.1	651	32.9
Pus/abscess	368	15.1	30	6.6	338	17.1
Upper respiratory tract	283	11.6	0	-	283	14.3
Urine	232	9.5	70	15.5	84	4.2
Blood	201	8.3	28	6.2	73	8.7
Lower respiratory tract	109	4.5	25	5.5	84	4.2
Eye discharge	95	4.0	5	1.1	90	4.5
Ear	71	3.0	8	1.8	63	3.2
High vaginal swab	66	2.7	13	2.9	53	2.7
Cerebrospinal fluid	29	1.2	0	-	29	1.5
^d^Miscellaneous	54	2.2	1	0.2	53	2.7
Total	2430	100	451	100	1979	100

^a ^Methicillin-resistant *Staphylococcus aureus*; ^b ^Methicillin-sensitive *Staphylococcus aureus*; ^c ^*P*eritoneal dialysate, 3; hemodialysate, 6; pericardial fluid, 4; pleural fluid, 7; joint fluid, 9; umbilical swab, 25;

The antimicrobial susceptibility patterns of MSSA isolates are shown in Table 2. Throughout the study period resistance to the older β-lactam antibiotics increased significantly (p < 0.001) in both hospital and community practices. Resistance patterns of erythromycin, co-trimoxazole, gentamicin and clindamycin were higher among hospital isolates when compared to community isolates, but these differences (except for gentamicin) were not statistically significant. The reverse situation was seen among community strains versus hospital strains where resistance rates were considerably higher for ciprofloxacin.

**Table 2:** Percentage of 1979 ^a^MSSA strains resistant to various antimicrobial agents at the Eric Williams Medical Sciences Complex, 1999 – 2004

Antimicrobial	Hospital Practice (n = 1375)	Community Practice (n = 604)	Significance (P-value)
Ampicillin	1356 (98.6)	412 (68.2)	< 0.001
Tetracycline	587(42.7)	444 (73.5)	< 0.001
Erythromycin	198 (14.4)	37 (6.1)	NS
Clindamycin	144 (10.5)	50 (8.3)	NS
^1^Co-trimoxazole	140 (10.2)	26 (4.3)	NS
Gentamicin	268 (19.5)	7 (1.2)	< 0.001
Ciprofloxacin	28 (2.0)	45 (7.5)	NS
Oxacillin	0	0	__
Vancomycin	0	0	__

^a^Methicillin-sensitive *Staphylococcus aureus*; ^1^Trimethoprim-sulfamethoxazole.

All MRSA isolates were fully sensitive to vancomycin, while the greatest prevalence of resistance was seen for erythromycin (86.7%) followed by clindamycin (75.3%), tetracycline (78.7%) and ciprofloxacin (59.1%). The remaining isolates had varying degrees of resistance which accounted for < 45%. Comparison of non-β-lactam resistant MRSA of the current study with a previous study is shown in Table 3. Resistance rates to erythromycin and ciprofloxacin were significantly higher when compared to the 1999 study (p < 0.001). Conversely, there were significant improvements in susceptibility among MRSA strains to tetracycline, gentamicin and co-trimoxazole when compared to the 1999 report [21]. This observation is encouraging since these drugs are among the cheapest on the hospital formulary. Interestingly, these drugs are also infrequently used (except gentamicin) on the wards (unpublished data). However, these statistical comparisons may not have been valid if the number of isolates for both studies were similar. The ratio of MRSA in the present study when compared with the previous study was approximately 10:1. The overall methicillin-resistance rate was 18.6%. Among hospital strains of MRSA, the rate of isolation was 20.8%, whereas, the prevalence of resistance among community isolates was 8.1% (Table 4).

**Table 3:** Comparison of the current resistance patterns of ^a^MRSA with a previous study at the Eric Williams Medical Sciences Complex

Antimicrobial	Current Study 1999 – 2004 (n = 451)	Previous Study 1997 – 1998 (n = 44)	P-value
Erythromycin	86.7	68.2	< 0.001
Tetracycline	78.7	93.2	< 0.001
Gentamicin	44.7	61.4	< 0.001
^1^Co-trimoxazole	13.3	45.4	< 0.001
Ciprofloxacin	59.1	20.5	< 0.001
Chloramphenicol	17.3	_	_
Clindamycin	75.3	-	-

^a^Methicillin-resistant *Staphylococcus aureus*, ^1^Trimethoprim-sulfamethoxazole.

**Table 4:** Distribution of 2430 *Staphylococcus aureus *strains isolated from hospital practice and community practice at the Eric Williams Medical Sciences Complex, according to methicillin-resistance, 1999 – 2004.

Methicillin	No. of hospital isolates (%)	No. of community isolates (%)	Total (%)
Sensitive	1581 (79.2)	398 (91.9)	1979 (81.4)
Resistant	416 (20.8)	35 (8.1)	451 (18.6)
Total	1997 (100)	433 ((100)	2430 (100)

Hospital MRSA: 1998 versus 2004, X^2 ^= 2.28; P = NS; Community ^b^MRSA: 1998 versus 2004, X^2 ^= 2.48; P = NS.

## Discussion

The present study has shown a steady increase in the prevalence rate of MRSA isolation (18.6%) over the previous study of 9.8%. Similar patterns have been seen worldwide as evident from the many recorded surveillance studies [3,4,18]. Despite this observation however; there are considerable differences between individual countries [22]. The highest rates of methicillin resistance among *S. aureus *were observed among isolates from the western Pacific region [15]. In Korea, the rate was 64 % [22], in a major Taiwanese hospital resistance rate was found to be about 77% [23]. In North America [4,24,25], the Middle East [7,9,13,26], India [27] and the Canary Islands [28] rates of 38 – 50%, 33–40%, 18 – 43% and 25% respectively, have been reported. MRSA rates from the Caribbean are scanty, but data from a previous report [21], Jamaica [29], Cuba [30] and a French territory island [31] have shown rates of isolation to be < 10%. From South America [4,32-34] and Europe [18,35,36], rates of 10 – 66% and 25 – 58% respectively, have been seen. These different rates among MRSAs from different countries maybe attributed to variations in patient populations, the biological characteristics of the *S. aureus *strains, and/or infection control practices.

The current study showed that the epidemiology of MRSA at this institution has changed. Although MRSA remains largely a nosocomial pathogen, 8.1% of isolates were community-acquired. Several factors maybe at play in the increasing prevalence of community-acquired MRSA. One reason for this increase maybe lateral dissemination of MRSA from hospital to the community from discharged patients diagnosed with MRSA, and the discontinuation of therapy because of the high cost of prescribed drugs at local pharmacies. These strains of MRSA are frequently multi-resistant. Two reports from Australia suggests that while lateral dissemination from hospital to community may be a major factor, strains of MRSA isolated from people in a remote western area of the country shows no similarity to hospital-derived strains. These people have had little or no contact with urban cities, health care facilities or health care providers, and these MRSA are generally non-multi-resistant [37,38]. The authors used multi-locus sequence typing and protein A gene typing, as well as susceptibility profiles in evaluating relatedness of the community-acquired MRSA strains. Although molecular techniques were not applied in this study, the multi-resistance as seen among community strains would suggest that these strains may have originated from the hospital.

The methicillin resistance rate of 20.8% as seen among hospital isolates was considerable higher than the 1999 rate of 12.5%. Community MRSA prevalence remained relatively stable over the past 6 years (4.1% in 1999 to 8.1% in 2004), but this was not statistically significant. These increases as seen in the study are certainly a cause for concern in Trinidad and may very well represent a trend in the changing epidemiology of MRSA in the 2000s. Although a protocol exists for the management of infected patients and colonized health care providers (HCPs), it is rarely implemented. The protocol requires the physical separation of infected patients and their HCPs from uninfected patients, therapy for eradicating MRSA, stringent environmental disinfection of areas harboring MRSA and the application of barrier isolation precaution measures, including strictly enforced hand hygiene to interrupt spread patterns. A previous report from this country revealed that proper hand washing practices and sanitation techniques of HCPs at all levels of service were not strictly adhered to [39]. The situation is further complicated by the fact that HCPs frequently complain of overcrowded wards, scarce material resources and overworked personnel.

## Conclusion

The study has shown that the prevalence of MRSA infections has increased over the years. The principal source may have been the hands of HCPs during wound dressing exercises. Infection control measures such as proper hand hygiene and surveillance cultures may help in arresting the spread of MRSA in the hospital setting. An antibiotic policy and the monitoring of susceptibility patterns of MRSA may also help in decreasing the prevalence of MRSA and antibiotic resistance.

## Competing interests

The author(s) declare that they have no competing interests.

## Authors' contributions

FAO planned the study, collected the data, analyzed the data, compiled the results and wrote the manuscript. ML helped in planning the study, analyzing the culture and susceptibility data and the statistical analysis.

## Pre-publication history

The pre-publication history for this paper can be accessed here:


